# Supplementary material for: Are carbon emissions trading and green financial instruments synergistic? -Comprehensive quantitative research based on content analysis
Source: PLoS One. 2024 Mar 7;19(3):e0298601. doi: 10.1371/journal.pone.0298601 (PMC10919601; doi:10.1371/journal.pone.0298601)
Supplement: S1 Data — (ZIP) [file pone.0298601.s001.zip › Data/地级市Z_F工作报告.zip!/║■─╧/│ú╡┬╩╨/└┤╫╘╣1⁄2╓┌║┼í╛┬φ┐╦╩2╛▌═°í┐_2017.docx]

各位代表：

现在，我代表市人民政府，向大会报告政府工作，请予审查，并请各位政协委员和其他列席人员提出意见。

一、过去四年及2016年工作回顾侯卫东官场笔记

市六届人大一次会议以来的四年，是常德发展历程中极为重要、极具挑战、极不平凡的四年。面对复杂的形势和艰巨的任务，全市人民在市委的坚强领导下，适应新常态，善于新作为，敢于新突破，集中打好“三大战役”，奋力开启新常德新创业的宏伟征程，实现了经济社会发展的新跨越。四年来，以“三改四化”、海绵城市试点为重点的城市建设取得突破性进展，以文化旅游、美丽乡村、完美社区为重点的美丽经济建设取得突破性进展，以引进战略投资者、园区开放开发为重点的产业建设取得突破性进展，以高速公路、城市出城口、沅澧快速干线、4D级机场为重点的交通建设取得突破性进展，以脱贫攻坚、城乡饮水安全全覆盖和教育卫生三年攻坚为重点的民生建设取得突破性进展，常德人民对我们共同生活的这座城市认同感越来越高，归属感越来越强，自豪感越来越足。

这四年，我们千方百计稳增长，经济实力再上台阶。地区生产总值由2012年2038亿元增加到2016年2900亿元（预计数，下同），总量居全省第三，年均增长9.4%。三次产业结构由14.8：49.5：35.7转变为13：44：43。完成固定资产投资1980亿元，一般公共预算收入227亿元，分别是2012年的2倍和1.5倍。新型工业化步伐加快。大力推进“1115”工程，全市亿元企业达到505家，百亿产业达到8个，百亿园区达到7个，规模工业增加值突破千亿大关；高新技术企业达到114家，高新技术产业增加值达到270亿元，是2012年的1.8倍。农业现代化推进有力。五个“双百亿”产业初步形成，大宗农产品生产保持稳定，全市农产品加工销售收入突破千亿大关，创造了农业标准化建设“六个一”常德模式，成功创建西湖西洞庭和桃源国家现代农业示范区。现代服务业发展强劲。大力发展美丽经济，打造了欢乐水世界、柳叶湖环湖景观、穿紫河风光带等一批旅游热点，旅游总收入达到318亿元，是2012年的2.4倍。着力打造六大城市商圈和五大物流园，覆盖全市、辐射周边的商贸物流体系基本形成。民生银行、广发银行等20家金融机构落户常德，成功组建常德农商银行和各县市农商银行，益丰药房、惠生国际成功上市，12家企业在新三板挂牌。社会消费品零售总额迈上千亿台阶。县域经济来势较好。桃源县、鼎城区、澧县、汉寿县、石门县地区生产总值超过200亿元，武陵区、鼎城区、常德经开区、桃源县一般公共预算收入超过15亿元。

这四年，我们全力以赴抓建设，城乡面貌焕然一新。城市建设力度空前。编制实施总体城市设计，市城区建成区面积扩大到93平方公里，人口增加到90万，城镇化率提升到49.3%，比2012年提升6.3个百分点。“三改四化”新建改造骨干道路和大街小巷310条280公里，铺设地下管线1100公里，完成棚户区改造11.5万户，建成“三馆三中心”、白马湖公园、丁玲公园、武陵阁步行城等一批城市新亮点，城市绿化、亮化、美化、数字化走在全省前列。成功跻身全国海绵城市建设试点城市、国家智慧城市建设试点城市、国家森林城市和全国绿化模范城市。县市城区提质扩容掀起高潮，获得了一批国家级省级荣誉。农村面貌大为改观。建成省级美丽乡村示范村14个、市级美丽乡村示范村57个。新增设施农业和高效田土7.2万亩，实施土地整治135万亩，建设高标准农田60万亩。除险加固小型水库867座，建设集中供水工程440处，在全省率先实现农村饮水安全全覆盖。完成市县城区防洪圈建设，成功应对沅水流域超历史洪水。推进农村环境综合整治，大力实施农村垃圾、畜禽水产养殖污染、农业面源污染治理和农村水源保护，农村环境质量大幅提升。基础建设加快推进。累计投入交通建设资金248亿元，新建高速公路226公里，新改建干线公路600公里，石长铁路复线、常岳高速、东常高速、常安高速建成通车，4D级桃花源机场投入使用，市城区80公里高速环线全线贯通，实现了城市与高速公路、空港“八进八出”的快速对接。常德电厂、桃源水电站建成发电，“五县一市”天然气输配管网工程全面竣工。

这四年，我们尽心竭力惠民生，人民群众普遍受益。全市民生支出累计投入1300亿元。城乡居民人均可支配收入分别达到26400元和12700元，是2012年的1.3倍和1.6倍。新增城镇就业27.5万人、农村劳动力转移就业31.4万人。城乡居民基本养老保险、医疗保险基本实现法定人员全覆盖，企业退休人员基本养老金实现“12连增”，累计支付社会保险待遇318.2亿元。城乡低保实现应保尽保，低保标准大幅度提高，新建改扩建各类养老机构302个。教育卫生三年攻坚成效明显。新建改扩建各类学校965所，建成合格学校600所，提前实现普及高中目标，成功申办湖南幼师高专和常德技师学院，湖南应用技术学院实现专升本，职教大学城初具规模，全市高考文科总分平均分连续5年全省第一，理科总分平均分连续7年全省第二；新建改扩建医疗卫生项目932个，市第一人民医院主楼等项目投入使用，湘雅常德医院即将运营。脱贫攻坚扎实推进。突出驻村帮扶、结对帮扶和“五个精准到户”，实施“六件实事”攻坚行动，26.6万建档立卡贫困人口实现脱贫。生态环境持续改善。深入开展蓝天碧水净土行动，空气质量优良率保持较好水平，城市饮用水水源地水质全部达标，农村主要水体水质持续好转，森林覆盖率达到47.98%。社会治理能力提升。深入推进平安常德建设，普法工作成效显著，社会治安综合治理不断加强，刑事案件发案率持续下降，信访秩序持续好转，安全生产形势持续稳定，食品药品安全监管水平有效提高，各类应急突发事件得到妥善处置，人防、气象、地震等防灾减灾体系进一步健全。各项事业全面进步。获得国家科技进步先进市称号。市非物质文化遗产展示厅、丁玲纪念馆等文化设施建成开放，“百团大赛”等群众性文体活动蓬勃开展，里约残奥会荣获两金一银。残疾人工作获得全国先进。“单独两孩”“全面两孩”、计生奖励救助等政策有效落实。国防动员、国防教育和民兵预备役工作切实加强，荣获全国人防工作先进单位、全国双拥模范城市称号。统计、粮食、住房公积金、民族宗教、对台、侨联、老龄、方志、档案等工作取得新成绩。工会、共青团、妇联为常德发展作出新贡献。

这四年，我们攻坚克难促改革，发展活力充分显现。重点领域改革实现新突破。聚焦中央和省委部署的重大改革事项，完成新一轮政府机构改革，稳妥推进事业单位分类改革，乡镇区划调整全面完成，文化体制综合改革、土地确权登记颁证等工作进入全国先进行列，商事制度、不动产统一登记制度、投融资体制、医药卫生体制、公路管理体制等改革取得明显成效，公车改革稳步推进。聚焦发展难题和瓶颈制约，市里自主推进的工业园区体制改革稳步实施，城市管理体制进一步理顺，基层社会治理体系创造常德模式，完美社区建设经验在全国全省推介。开放合作呈现新局面。突出承接对接和战略合作，先后与中国医药集团、中国兵装集团、中国建材集团等10多家央企、省企签订战略合作协议，成功引进华侨城、万达、保利、浪潮等战略投资者。累计引进内外资总额2361亿元，完成进出口总额201亿元。盐关码头国家二类口岸完成升级改造，我市正式进入内陆口岸城市行列。新增1个国际友好城市、1个国际友好合作关系城市。

这四年，我们公开透明重监督，民主法治全面加强。坚持把政府工作纳入法治轨道之内，置于严格监督之下。自觉接受市人大及其常委会的法律监督和工作监督，定期向人大报告工作，认真执行解决城乡居民安全饮水问题等决议决定，积极处理各项审议意见，主动报送规范性文件备案审查，为人大及其常委会依法监督、人大代表视察检查活动创造条件。积极支持市政协履行政治协商、民主监督和参政议政职能。虚心听取民主党派、工商联、无党派人士和人民团体的意见建议。办理人大代表建议931件、政协提案1546件。坚持科学民主决策，把公众参与、专家论证、风险评估、合法性审查、集体讨论决定作为政府决策的必经程序，建立实施重大行政决策听证、政府法律顾问、政府合同管理等制度，切实加强政务公开，着力完善立法机制，扎实推进行政执法规范化建设。

这四年，我们坚持不懈优服务，行政效能显著提升。转变政府职能，着力打通服务群众的“最后一公里”。简政放权力度不断加大。全面清理行政审批事项，编制公布“三清单一目录”，强力推进“六个压减”，“三公”经费压减41.7%、行政审批事项压减34.5%、审批时限压减64%、市级权力事项精简26%。加强社会求助服务、公共资源交易、网上政务服务等平台建设，创新性编制《政策清单》，群众办事更加便捷。经济发展环境不断优化。严格实行优化经济环境十项禁令，大力整治中介服务、工程建设和办事服务环境。落实党风廉政建设“两个责任”，坚持纠“四风”治陋习，加强行政监察和审计监督，查处了一批违法违纪人员。扎实开展党的群众路线教育实践活动、“三严三实”专题教育和“两学一做”学习教育，严格落实改进作风各项规定，政风行风明显好转。

各位代表，我们共同走过的这四年，有目标、有奋斗、有艰辛，不容易、不简单、不平凡。四年来，我们在市委的坚强领导下，励精图治，奋发图强，形成了新常德新创业的常德共识，彰显了改革创新的常德担当，表现了攻坚克难的常德作为，展现了防控风险、破解瓶颈的常德智慧，凝聚了团结奋斗的常德力量，塑造了风清气正的常德形象。此时此刻，我们由衷地为这座城市点赞，为善良勤劳智慧的常德人民点赞，为忠诚干净担当的广大党员干部点赞。光荣和梦想永远属于常德人民！

刚刚过去的2016年，是“十三五”的开局之年，是市县乡三级同步换届之年。我们积极应对经济下行压力加大的严峻形势，认真贯彻中央和省委省政府推进供给侧结构性改革等重大决策部署，持续推进民生升温、园区攻坚、城市提质三大战役，经济社会发展稳中有进、稳中向好。地区生产总值增长8%，规模工业增加值增长6.5%，固定资产投资增长14.5%，一般公共预算收入增长9.1%，城乡居民人均可支配收入分别增长7.8%、8.6%。

一年来的主要工作和成效是：

1、持续推进园区攻坚，产业发展来势看好。着力夯实园区平台，完成工业园区基础建设投入75亿元，建设标准化厂房160万平方米，国家级高新区创建进入候批程序，国家农业科技园区创建取得新进展，津市工业集中区获批为省级高新区。着力狠抓重大项目，烟厂易地技改、中联重科起重机械产业基地等项目加快建设，中锂二期、昊晖光伏、晋煤金牛等项目建成投产，全年引进内外资总额680亿元，开工建设亿元以上项目257个。着力帮扶企业发展，开展“千名领导干部联系服务千家企业”活动，出台帮扶工业企业十条，市财政安排4亿元工业企业帮扶解困专项资金，为企业提供过桥贷款5.8亿元，提供融资担保和小额贷款等65亿元；支持促进创新创业，新增市场主体3.6万户，新增农民合作社674个、家庭农场1389家、规模农产品加工企业30家。着力壮大美丽经济，桃花源景区主体工程及其配套建设基本完成，老西门文化旅游街区开业，常德河街开盘，德国风情街开街，穿紫河水上观光旅游开航，华侨城卡乐世界开工，城头山遗址公园开园，万达广场、友阿国际、和瑞欢乐城等大型城市综合体开张营运；成功举办常德旅游节、常德柳叶湖国际马拉松赛，乡村旅游丰富多彩，太阳谷入选中国最美休闲乡村。

2、持续推进城市提质，基础建设实现突破。不断提升城市功能品质，迎宾大道、柳叶大道西延线、阳明大道建成通车，沅江隧道开工建设，江南风光带建成开放，城市公交优先三年行动全面启动。扎实推进海绵城市建设，投入资金20多亿元，全面整治城市黑臭水体，实施海绵城市建设项目76个。加快交通建设攻坚步伐，完成投资95亿元，创历史新高，新改建干线公路151公里，新建改造农村公路1053公里，完成危旧桥改造182座，黔张常高铁建设进展顺利，沅澧快速干线推进有力，319、207国道市城区改线工程加快建设，德山港区和澧县戴家湾码头基本建成。大兴水利基础设施建设，完成一线大堤除险加固140处、小型农田水利工程3万多处。深入开展智慧城市建设，依托云计算中心，加快构建数据资源共享平台，智慧城管、智能交通、数字防控、社会治理网格化管理等系统不断完善，石门云数据中心建成运行。

3、持续推进民生升温，群众福祉不断改善。扎实开展教育卫生攻坚，改造农村薄弱学校139所，新建改扩建城区中小学165所，建成公办和普惠性民办幼儿园68所，常德技师学院新校区建成投入使用；新建改扩建医疗卫生项目343个，市第二人民医院和石门县、澧县、汉寿县人民医院创建为三级医疗机构，常德被评为全国基层中医药工作先进市。大力推进脱贫攻坚，确定兜底保障对象2.7万人，开工建设易地搬迁项目59个，实现8万人脱贫。积极促进就业创业，新增城镇就业6.7万人、农村劳动力转移就业4.1万人，新增创业主体2.7万户，五类保险新增参保19.1万人。切实加强环境保护，洞庭湖水环境综合治理五大专项行动全面开展，大气污染防治和土壤污染综合治理扎实推进，“三山”保护工作全面启动，津市毛里湖创建成为国家湿地公园。不断创新社会治理，“3+N”社区治理模式和网格化治理机制全面推行，农村社会治理创新试点范围不断扩大，社会治安综合治理切实加强，社会大局和谐稳定。

各位代表！

在过去的四年里，我们取得的每一点成绩和进步，都是市委统揽全局、正确领导的结果，是市人大和市政协有效监督、大力支持的结果，是全市人民和社会各界齐心协力、共同努力的结果。在此，我代表市人民政府，向全市人民，向各位人大代表、政协委员，向各民主党派、工商联和社会各界人士，向驻常人民解放军、武警、消防官兵和政法干警，向关心支持常德发展的同志们、朋友们，表示衷心的感谢！

成绩令人鼓舞，问题不容忽视。我们在前进道路上还面临不少挑战与困难，政府工作还存在不少差距和不足，主要是：经济下行压力较大，实体经济、工业经济、中小微企业发展困难较多，财政收支平衡面临压力，部分经济指标没有完全实现预期目标。增长动力接续不足，传统支柱产业拉动作用明显减弱，新兴产业尚未形成足够拉动力。县域经济、非公经济、开放经济、金融经济仍然是发展的薄弱环节。脱贫攻坚任务繁重，城乡公共服务与人民群众的需求还有差距。作风建设仍需加强，一些部门和单位服务意识不强、行政效能不高、工作落实不到位，少数干部不会为、不作为、慢作为甚至乱作为，消极腐败现象还不同程度地存在。对这些困难和问题，我们一定高度重视，勇于担当，以民之所望为政之所向，努力做好政府工作，决不辜负组织的重托，决不辜负人民的期望！

二、今后五年政府工作的总体要求和目标任务

今后五年，是决胜全面小康开启现代化新征程的攻坚时期，是适应新常态培育新动能的转型时期，是立足新起点推进新常德新创业的关键时期。当前，我们面临复杂多变的外部形势。世界格局深刻调整，经济复苏存在不确定性，我国经济面临较大的下行压力，经济增速换挡、结构调整阵痛、新旧动能转换相互交织，我们必须因势而谋、积极应对，在复杂形势中把握发展主动。我们面临机遇叠加的有利趋势。中央稳中求进的工作总基调没有变，国家实施积极财政政策和稳健货币政策的宏观政策取向没有变，我国经济长期向好的基本面没有变，供给侧结构性改革的效应逐步显现，“一带一路”、长江经济带、洞庭湖生态经济区和促进中部崛起新十年规划等国家战略加快实施，我们必须应势而动、抢抓机遇，在有利趋势中赢得发展先机。我们面临蓄势待发的前进态势。新常德新创业的推进实施，给沅澧大地注入了蓬勃生机和巨大活力，一大批重点项目相继开工、建成受益或取得实质性进展，常德发展的能量正加快释放，我们必须乘势而上、后发赶超，在前进态势中加快发展步伐。我们面临政通人和的发展来势。今天的常德，干事创业的氛围非常浓厚，未来的常德，跨越发展的预期普遍看好，我们必须顺势而为、奋发进取，倍加珍惜发展的大好局面，奋力实现发展的美好蓝图。

根据新的形势和任务，政府工作的总体要求是：紧密团结在以习近平同志为核心的党中央周围，深入贯彻习近平总书记系列重要讲话精神，全面落实省市党代会的工作部署，以“五位一体”总体布局和“四个全面”战略布局为引领，以提前全面建成小康社会为总揽，坚持创新、协调、绿色、开放、共享发展新理念，着力推进供给侧结构性改革，主动适应引领经济发展新常态，围绕“一三五”的发展战略目标，深入推进新常德新创业，为建设现代化区域中心城市而努力奋斗。

按照这一总体要求，今后五年我市经济社会发展的主要目标任务是，打造“一个中心”，实现“三个迈进”，建设“五个常德”，强力推进“四大会战”，推动经济更高质量、更有效率、更加公平、更可持续的发展。打造“一个中心”，就是要努力把常德打造成泛湘西北现代化区域中心城市。实现“三个迈进”，就是要迈进高铁时代、迈进大城市时代、迈进千亿产业时代。建设“五个常德”，就是要坚持创新发展、建设智慧常德，坚持协调发展、建设健康常德，坚持绿色发展、建设美丽常德，坚持开放发展、建设现代常德，坚持共享发展、建设幸福常德。推进“四大会战”，就是要推进脱贫攻坚大会战、交通跨越大会战、园区攻坚大会战、城镇带动大会战。今后五年，地区生产总值年均增长8%，规模工业增加值年均增长8%，固定资产投资年均增长15%，一般公共预算收入年均增长10%，城乡居民收入年均增长9%，产业结构更加合理，基础设施更加完善，公共服务更加优化，各项保障更加健全，社会安定有序，人民安居乐业，提前全面建成小康社会。

通过五年努力，奋力夺取新常德新创业的新成就，开创新常德新创业的新辉煌。

1、建成交通枢纽，再造区域发展新优势。完善航空航线布局，打造空港物流基地；实现黔张常、长益常高铁建成通车，宜常高铁、常岳九铁路开工建设，呼南、厦渝高铁在常德交汇，成为高铁“新贵”城市；建成益常高速复线、安慈高速，实现县域高速公路全覆盖；完成沅澧快速干线建设，形成市域内快速通勤圈；完成沅澧航道、淞虎航道系列整治工程，积极打造常德水运新优势。基本形成高速、高铁、航空、水运一体化，国省干道和县乡村公路网络化的现代交通运输体系，基本建成区域性交通枢纽城市。

2、建成千亿产业，壮大经济跨越新动能。把烟草、生物科技与健康食品、现代装备制造业培育成过千亿的支柱产业，确保建成德山千亿园区，力争建成常德国家级高新区和津澧国家级经开区；加快发展文化旅游产业，培育壮大美丽经济，确保桃花源建成国家5A级景区、柳叶湖建成国家级旅游度假区，把常德打造成国内外知名的文化旅游、休闲度假目的地。形成以现代农业为基础、战略性新兴产业为先导、先进制造业为主导、现代服务业为支撑的产业格局，为经济跨越打造强力引擎，注入强劲动能。

3、建成双百城市，构建城镇带动新格局。市城区建成区面积超过100平方公里、人口超过100万，迈进大城市时代，让城市功能更完善、品质更精美、品位更时尚；建成沅江隧道、沅水四桥等重点项目，加快北部新城、东部城区、江南新城、德山新城等片区建设，实现“一江两岸”“一体三极”协调发展；强力推进澧县、津市合并设市，加快常桃融合，构建津澧、桃源副中心，加快县城城市化，因地制宜建设一批工业强镇、商贸重镇、文化旅游名镇等特色小镇，全市城镇化率达到55%以上，实现城市人口超过农村人口的历史跨越，走出一条中心城市+副中心城市+城镇群的新型城镇化发展路子。

4、建成开放高地，激发创新创业新活力。形成开放的发展平台，构建完善的开放口岸，建成常德保税物流中心；形成开放的发展环境，促进各类市场主体公平竞争，促进大众创业万众创新；形成开放的经济发展格局，引进战略投资者实现新的突破，累计引进内外资总额4500亿元。通过五年努力，把常德建成现代化的开放发展高地，努力实现开放崛起。

5、建成全面小康，谱写幸福和谐新篇章。坚决打赢脱贫攻坚战，在2018年实现全市农村贫困人口整体脱贫，提前实现全面小康；水、大气、土壤等环境质量进一步优化，全市空气质量优良率达到82%以上，森林覆盖率稳定在48%左右，巩固提升城乡饮水安全全覆盖成果，让人民群众喝上放心水、安全水；城乡公共服务更加完善，社会保险基本实现应保尽保，城镇登记失业率控制在4.5%以内，人民群众精神文化生活更加丰富，社会更加安全稳定和谐，发展成果更多更公平地惠及全体人民。

三、2017年工作安排

2017年是新一届政府的开局之年。我们肩负的发展和改革任务十分繁重，必须以中央、省委、市委经济工作会议精神为指针，牢牢把握稳中求进的工作总基调，坚持以提高发展质量和效益为中心，以推进供给侧结构性改革为主线，扎实推进脱贫攻坚、交通跨越、园区攻坚、城镇带动四大会战，奋力开创新常德新创业的新局面。经济社会发展的主要预期目标是：地区生产总值增长8%以上，规模工业增加值增长7%以上，固定资产投资增长15%以上，一般公共预算收入增长10%以上，城乡居民人均可支配收入增长8%以上，完成省里下达的节能减排目标任务。围绕实现以上目标，重点抓好十项工作：

1、大力推进工业创新升级

坚持创新引领战略，把工业作为供给侧改革的主战场，努力促进工业经济规模和质量“双提升”，年内新增规模工业企业100家以上。

加强平台建设。积极推进园区攻坚大会战。支持常德经开区创建国家级新型工业化产业示范基地，促进西洞庭生物科技园、津市生物医药产业园等工业园区向特色化、专业化、集约化方向发展。加大标准化厂房和配套设施建设力度，完成园区基础设施投入80亿元以上。完善“一权两制一司”运行机制，提升园区自主发展能力。力争新增1个百亿园区，确保常德高新区成功创建国家级高新区，支持有条件的区县工业集中区创建省级高新区。

促进产业升级。全面对接“中国制造2025”和湖南省建设制造强省五年行动计划，启动“两化”融合试点示范工作。探索设立产业基金，推动构筑新兴优势产业链。加快烟草、酿酒、食品、装备制造、有色金属等传统产业升级改造，全年完成技改投入500亿元以上。大力发展新材料、新能源、电子信息、生命科学等战略性新兴产业，加快烟厂易地技改、中联重科起重机械产业基地、智慧产业园、生命科学与健康产业园等重点项目建设。做大做强骨干企业，培育壮大中小微企业，新增亿元企业10家以上，“1115”工程企业产值突破一千亿元。

实施创新驱动。强化企业创新主体地位，支持企业争创国家和省级重点实验室、工程技术研究中心，建设院士专家工作站、创客中心和创新孵化基地，大力引进创新型人才，新增省级科技企业孵化器1家以上、省级众创空间1家以上。完善厅市合作、校企合作机制。实施一批重大科技专项，完成科技成果转化项目20项以上。新增高新技术企业10家以上，高新技术产业增加值增长15%以上，发明专利申请量和授权量增长12%以上。积极开展质量品牌行动，大力实施标准化战略，弘扬企业家精神和“工匠精神”，支持企业创建名品名牌。

强化企业帮扶。全面落实帮扶工业企业十条等政策措施。加强政银企衔接协调，更好发挥担保公司、投资公司及信贷过桥资金作用，着力解决企业需求最迫切的融资问题；健全落实降低涉企中介收费、降低水电气生产成本等政策措施，着力解决企业反映最急切的成本问题；加大工业企业技术改造、兼并重组奖励支持力度，着力解决企业发展最关切的转型问题；鼓励推广使用地方名优产品，深入推进“千名领导干部联系服务千家企业”活动，构建“亲”“清”新型政商关系，着力解决企业感受最深切的服务问题。当前，实体经济、中小微企业正处在爬坡过坎的关口，企业面临的问题就是政府的课题，企业家的难处就是政府的用心处，各级政府及其相关部门，在企业的发展上要积极有为地和企业家站在一起、想在一起、干在一起，以最大的诚心、最大的毅力、最大的力度动员资源，聚集能量，解决问题，共同孕育实体经济的春天。

2、大力推进现代农业发展壮大

加强现代农业产业体系建设。大力推进农业结构调好调顺调优，推行绿色生产方式，以绿色供给满足绿色需求。稳定发展粮食产业，确保粮食安全；转型发展生猪、水产等养殖业，加快发展蔬菜、林业、油茶、乳业等高效产业，提升五个“双百亿”产业水平。加快国家农业科技园区和西湖西洞庭、桃源国家现代农业示范区建设，促进柑橘、茶叶等优势产业向优势产区集中，甲鱼、葡萄等特色产业向专业园区集中，新增省级特色农业产业园区15个。积极发展立体种养、休闲体验农业等新型农业业态，实施农产品加工业振兴工程，促进一二三产业深度融合发展。

加强现代农业生产体系建设。着力推进农业标准化，完善生产技术规程，开展农产品质量安全提升行动，加强农产品品牌创建、营销，培育和打响常德农副产品品牌。着力推进农业机械化，提高水稻等主要农作物全程机械化水平。着力推进农业信息化，大力发展智慧农业、精细农业。

加强现代农业经营体系建设。全面落实农村土地“三权分置”政策措施，完成农村土地确权登记颁证工作，稳步发展土地流转、土地入股等多种形式的适度规模经营。深入实施新型职业农民培育工程，培育壮大新型农业经营主体，新增家庭农场500家、农民合作社500个、规模农产品加工企业30家。

加强现代农业服务体系建设。推进农业科技创新应用，积极开展“互联网+”现代农业行动。推进常德农产品物流交易展示中心、农机大市场等流通设施和专业市场建设，搞活农产品流通。加快发展动植物疫病防控、农资配送等农业生产性服务业。落实强农惠农富农政策，强化农民负担监管。推进农村土地整治和农业综合开发，建设高标准农田15万亩。抓好防汛抗旱工作，减少农业灾害损失。

3、大力推进服务业繁荣扩张

以服务业繁荣带动消费扩张升级，加快打造泛湘西北区域消费中心。社会消费品零售总额增长12%以上。

做大文化旅游业。坚持把文化旅游作为战略性产业来抓，进一步培育壮大美丽经济。突出核心景区建设，确保桃花源景区7月盛大开园，完成华侨城卡乐世界等项目主体工程，积极支持城头山申报世界文化遗产，加快壶瓶山、花岩溪、林伯渠故居等提质升级。突出品牌形象提升，推进大湘西旅游经济协作区整体联动，积极融入湖南旅游“金三角”，完善旅游公共服务体系，加强宣传策划营销，办好常德旅游节、常德柳叶湖国际马拉松赛等活动，积极培育城市旅游、乡村旅游、红色旅游新热点，支持石门县创建国家全域旅游示范县，大力推动风景名胜区建设与申报，积极开展国家公园申报，促进“点上旅游”向“全域旅游”转变。突出文化旅游融合，加快文化创意产业发展，壮大游戏动漫、影视传媒、演艺娱乐、休闲会展等文化产业，打造一批文化旅游亮点。

做强现代金融业。支持常德农商银行和各县市农商银行做大做强，新引进1家股份制商业银行，支持商业银行机构网点下沉，培育一批村镇银行、小额贷款公司和融资性担保公司。激励金融机构开展产品和服务创新，扩大对实体企业、中小微企业的信贷投放，新增银行贷款150亿元以上、表外融资150亿元以上。鼓励企业创新融资模式和渠道，扩大债券发行规模，力争湘佳牧业中小板上市。支持保险业发展。推进社会信用体系建设，依法打击非法集资、金融欺诈等行为，规范融资平台管理，坚决防范区域性金融风险。

做活商贸物流业。推进白马湖、阳明湖商务商贸中心建设，进一步激活武陵阁步行城、万达广场、友阿国际、和瑞欢乐城、老西门、常德河街、德国风情街等商业区域的人气财气，引导商贸企业组织参与各类展会促销活动。建设一批大型物流园区，引进和培育一批物流龙头企业，促进中小物流企业整合发展，推动物流网络向乡村延伸。加强与龙头电商合作，扶持和培育本土电商示范企业，推动本地产品线上销售，完善农村电商服务体系。

做优其它服务业。推动研发设计、服务外包、节能环保、信息科技等生产性服务业向专业化和价值链高端延伸，促进住宿餐饮、家政服务、健康养老、健身休闲等生活性服务业向便利化、精细化、品质化发展。稳定房地产投资和供需，规范房地产市场秩序，推进拆迁货币化安置，积极化解房地产库存，培育发展存量房市场和住房租赁市场，促进房地产业平稳健康发展。

4、大力推进城镇更新提质

实施城镇带动大会战。加快城乡一体化发展，全市城镇化率达到50%以上。

提升规划建设水平。围绕宜居安居抓城建，围绕产城互动抓城建，围绕片区开发抓城建。完成城市总体设计编制报批，逐步推进“多规合一”，更好统筹各类空间规划。大力推进18个海绵城市建设重点项目，全面完成江北城区36平方公里海绵城市建设任务，打造技术示范基地和国际论坛“两大品牌”。积极推进市民中心、财鑫广场、东部城区提质改造、沅江隧道、丹洲生态城等重点项目建设。深入推进“三改四化”，抓好棚户区、城中村和老旧小区改造，完成棚户区改造5万户。进一步明确和发挥好中心城区区一级抓城市建设发展的工作责任。

增强综合承载能力。大力推进以人为核心的新型城镇化，加强城市公共交通、公共停车场所、便民服务网点等建设，深入实施城市公交优先三年行动计划；全面放开市中心城区落户限制，全面实施居住证制度，依法维护进城农民土地承包权、宅基地使用权和集体收益分配权，促进有能力在城镇稳定就业和生活的农业转移人口进城落户，让更多的农村人口融入城市。

完善城市管理工作。推进城市管理和执法体制改革，深化城市管理“一改四化”，更好发挥街道、社区作用，推进管理重心进一步下移。完善运营市场化管理方式，大力推行政府购买服务。规范提升住宅小区物业管理，规范道路停车管理，大力开展建设工地渣土、户外广告、交通秩序等专项整治行动，加强市民文明素质教育，巩固提升全国文明城市良好形象。

构建新型城镇体系。完善县城发展规划，支持县城提质扩容，稳步推进津澧融城，启动一批标志性融城工程，扎实开展国家中小城市综合改革试点和新型城镇化试点。实施“两西”发展战略，加快把西湖西洞庭建设成为独具魅力的常德卫星城。巩固乡镇区划调整成果，积极对接国家“千个特色小镇培育计划”，推进13个美丽城镇建设试点和新型城镇化示范项目建设，打造一批产业定位“特而强”、功能叠加“聚而全”、建设形态“精而美”、制度供给“活而新”的新型城镇。

5、大力推进基础设施配套完善

开展“项目推进年”活动，实施重点工程项目500个，完成投资1300亿元。

健全交通网络。全力推进交通跨越大会战。加快黔张常高铁建设，力争长益常高铁开工建设，做好宜常高铁、常岳九铁路前期工作。加快安慈高速建设，力争益常高速复线、宜石张高速项目开工建设。加快安乡长岭洲、桃源陬市、津市窑坡渡码头建设，完成沅水浦市至常德、澧水澧县至茅草街航道整治，启动鼎城蒿子港、汉寿岩汪湖码头建设。加快石门杨岭岗大桥、安乡长岭大桥、津市澧水二桥建设，确保安乡石龟山大桥建成通车，改造病危桥及渡改桥122座。推进沅澧快速干线建设，确保一号大道临岗段、二号大道石门段和临澧段、六号大道汉寿段建成通车，加快和启动四号、五号、六号、七号大道建设。加快推进319、207国道市城区改线工程等项目建设，新建改建国省干线公路113公里，新建改造农村公路1000公里。坚持办人民满意交通，实施交通行业“六项整治”，推进治超常态化，确保公路安全畅通。

完善水利设施。投入资金40亿元，重点推进江河湖库连通、大中型灌区续建配套与节水改造、大型泵站更新改造、一线大堤除险加固等工程。加强堰塘清淤扩容、渠道疏浚、机泵整修等小型农田水利建设。实施“两水四岸”综合整治工程，推进黄石水库水资源综合利用工程建设，全面推动农村饮水安全提质改造。

强化能源保障。加强城乡中低压配电网建设和农网升级改造。建成酒泉至湖南特高压输电线路常德段，加快500千伏常德北输变电、220千伏汉寿同心输变电等项目建设，加快岳阳至安乡、石门至张家界天然气管网建设，有序发展风力发电、光伏发电、地热能等清洁能源，积极推进多能互补。

建设智慧城市。加强政府数据资源共享，抓好“我的常德”城市公共服务平台建设，推进智慧教育、智慧医疗、智慧旅游等建设，完善智能交通、智慧城管、数字防控三大平台，加快社会治理网格化综合信息平台建设应用，建立统一的智慧常德协调指挥中心。支持武陵区创建国家智慧城市示范城区。启动中小城镇宽带提速工程，构筑城乡一体化信息高速公路。

6、大力推进县域经济做大做强

全力放权扩权。凡是已经明确下放到县的管理权限，一律下放到位；凡是明确取消的行政许可审批事项，一律不得变相审批；凡是有利于县域发展的管理和审批权限，一律能放尽放。扎实推进石门县省直管县体制改革试点、陬市镇全国经济发达镇行政管理体制改革试点等工作，科学合理划分市、县事权与支出责任，进一步明确县域发展的工作责任，增强争先进位意识，充分调动县域发展主观能动性，力争有更多县市进入全省县域经济二十强。

强力引导带动。加强对县域发展的规划和指导，积极谋划推进能够带动县域发展的重大产业项目、基础设施项目，提升市级引领发展、市县联动发展和县域自主发展水平。积极支持县市争取项目资金、争创各类试点示范、引进战略投资者。在政策、资金、土地等方面对县市给予倾斜，采取鼓励金融机构增加信贷投放、扩大市级担保机构担保支持、探索设立市县相关产业基金等方式，加大对县市产业发展、基础建设、环境整治等支持力度。

倾力关心基层。顺应合乡并村后的新情况，盘活利用闲置资产资源，抓好农村综合服务平台建设，加大乡村社区运转经费保障力度，保障好乡镇、村居干部待遇。关心基层干部成长，改善基层工作生活条件，鼓励优秀人才扎根基层建功立业。清理规范压缩面向基层的检查考核、评比达标活动，切实为基层减负解难。

7、大力推进生态环境保护利用

加强生态保护。科学划定和严守生态保护红线，实施生态修复、城市修补，统筹推进山、水、林、田、湖生态环境保护与修复工程。优先保护耕地，加强矿产资源管理和地质灾害防治。加大城乡生态创建力度，积极开展生态文明示范区建设，力争创建国家级生态文明建设示范乡镇15个以上。支持湿地公园建设。整市推进美丽乡村建设，加强农村环境整治和美丽庭院建设，建成美丽乡村20个以上。

强化环境治理。切实抓好大气污染防治，淘汰城市建成区内10蒸吨及以下燃煤锅炉和黄标车，加强移动污染源治理，市城区空气质量优良天数达到275天以上。认真开展洞庭湖水环境综合治理五大专项行动，加强各县市城区黑臭水体治理。加大农业面源污染、重点工业污染源、城镇生活污水防治力度，禁养区规模化畜禽养殖全部退出，所有公共水域禁止投肥养殖。强化水源保护和水质监测，全面推进饮用水水源保护区划定和规范化建设，推进工业园区和农村集镇污水处理厂及配套管网建设。启动土壤环境质量详查，加快土壤污染综合防治先行区建设。

促进绿色发展。完成重点行业落后产能淘汰，推进水泥、化工等重点污染行业清洁生产审核，加强企业清洁生产技术改造，全面落实节能减排任务。加强资源集约节约高效利用，加快发展循环经济，大力培育节能环保、新能源等新兴产业。大力推广低碳出行和绿色消费，引导生活方式和消费模式向节约、绿色、文明、健康的方向转变。

8、大力推进改革开放深化拓展

攻坚克难深化改革。全力推进中央和省里确定的改革任务，深入推进市里确定的自主改革，力争改革走在全省前列、形成常德特色。围绕“三去一降一补”扎实推进供给侧结构性改革，加快政府职能转变，推进“放管服”改革。深化行政管理体制、事业单位分类管理、农垦体制、综合行政执法、预算管理、税收征管、商事制度、供销社、国资监管体制等改革，加快医药卫生体制、公共资源交易管理体制改革。积极探索推进一批与发展紧密相连、与群众切身相关的改革，让人民群众在改革中获得实实在在的利益。

全力以赴扩大开放。狠抓区域合作升级，主动对接“一带一路”、长江经济带和洞庭湖生态经济区等国家战略，全面加强与沿海、沿江、沿湖地区的多层次协作，建设合作共赢的开放之城。狠抓口岸平台升级，完善盐关水运口岸设施，启动德山新港二期国际集装箱码头规划建设，积极申建桃花源机场临时航空口岸，加快常德保税物流中心建设。狠抓贸易提档升级，坚持外贸、外资、外经“三外联动”，内外贸互动发展，加强国家出口产品质量安全示范区建设，助推本地企业境外投融资和承揽工程项目，扩大对外劳务输出规模，大力培育外贸龙头企业和出口大户，全力推动“德企出海”。

千方百计招商引资。强化精准招商，以最大的决心和诚意引进一批战略投资者和外向型企业。提升专业化招商水平，整合共享招商引资信息，做好行业招商、商会招商、以商招商、跨区域招商、产业链招商、PPP项目招商、驻外机构招商，注重引老乡、回故乡、建家乡。坚持招商与招才并举、引资与引智并重，力争引进一个项目、带来一批人才、打造一个团队、搞活一个产业。注重招商实效，提高签约项目的履约率、资金到位率和开工率。力争引进亿元项目150个以上，引进内外资总额750亿元以上。

9、大力推进民生事业协调发展

强力推进脱贫攻坚。全力以赴打好脱贫攻坚大会战。深入推进“两个全覆盖”“五个精准到户”，切实办好新“六件实事”。实施精准建档和挂图作战，突出产业帮扶、金融帮扶、智力帮扶，激发脱贫攻坚的内生动力。建立健全贫困对象退出机制、资金项目监管机制和稳定脱贫长效机制，做好贫困人口动态管理，科学制定脱贫计划。确保石门县贫困县摘帽，全市贫困村退出150个以上，脱贫8万人以上。

积极促进就业创业。推动以创业带动就业，实施全民创业行动，鼓励引导广大民众积极创新创业、创造幸福生活。实施就业优先战略，抓好高校毕业生、农村转移劳动力、城镇就业困难人员和退役军人就业工作，支持灵活就业，发展新就业形态。完善城乡均等、功能齐全的就业服务体系，加强就业援助，推行劳动者终身培训制度，构建和谐劳动关系。新增创业主体2.7万户，新增城镇就业6.6万人、农村劳动力转移就业4万人。

完善社会保障体系。强化社保扩面，努力实现应保尽保。完善城乡医疗保险制度，减轻群众就医负担。提高城乡低保、特困供养保障标准和精准度，实现农村低保标准和扶贫标准两线融合。加快发展慈善事业，关心关爱留守儿童、孤寡老人和残疾人。

加快建设教育强市。改善办学条件，加快职教大学城、北部新城学校、市一中提质改造、市第一幼儿园分园新建等重点工程建设，加强农村偏远山区学校和薄弱学校建设，全面启动义务教育标准化学校建设，确保各区县市全部通过国家义务教育基本均衡县验收。提升教育质量，深入推进教育综合改革，探索教育集团化、联盟化发展，加强文明校园创建和师德师风建设，大力培育“名、优、特”教师。促进协调发展，加快公办和普惠性民办幼儿园建设，统筹义务教育一体化发展，促进高中教育优质特色发展，支持高等教育发展，做大做强职业教育品牌，抓好民办教育、特殊教育和老年教育。

切实保障人民健康。健全市县乡村四级医疗卫生服务体系，大力推进公立医院建设，做好湘雅常德医院运营管理，实施市第一中医院和中国医药集团合作整体迁建工程，加快建设省妇幼保健院常德医院。拓展国家基本公共卫生服务项目，加强传染病等重大疾病和公共卫生检测预警与防控，抓好重性精神障碍患者救治工作。扶持和规范社会办医，形成多元化办医格局。提升中医药服务能力。稳步实施“全面两孩”政策，加大出生缺陷综合防治和计生特困家庭帮扶力度，促进人口均衡发展。引导社会力量参与养老服务，探索建立以居家为基础、社区为依托、机构为补充、医养相结合的城乡养老服务体系。

协调发展各项事业。以文化自信为引领，进一步推进文化强市建设，完善现代公共文化服务体系，加强文化市场和行业监管，做好文化遗产保护，传承弘扬丝弦、大鼓、高腔等民间艺术，抓好文艺精品创作，加强文艺人才培育。推进奥体中心、第二工人文化宫等文化体育场馆建设，开展社区文化节等形式多样、丰富多彩的群众性文化活动。注重家庭家教家风，培育社会主义家庭文明新风尚。办好第七届市运会，广泛开展全民健身运动。促进广电事业发展。加强住房公积金归集和资金管理。做好统计、粮食、外事侨务、民族宗教、防震减灾、对台、方志、气象、档案、保密等工作。支持工青妇等群团组织工作。重视应对人口老龄化趋势，重视发展老龄事业。大力支持国防和军队改革，抓好国防动员、国防教育、军事设施保护、人民防空、预备役和征兵工作，加强双拥优抚安置等工作，促进军民融合深度发展。

10、大力推进社会治理规范有序

保障公共安全。积极创建国家安全发展示范城市，全面建立和完善安全生产责任体系，推进安全生产动态监控、隐患排查治理和应急指挥救援体系建设，严格安全生产监管执法，坚决杜绝较大及以上事故。全面落实食品药品网格化、信息化监管模式，大力推行“双随机”检查，抓好餐饮单位、学校、农贸市场食品安全的示范创建。深入开展平安创建活动，大力推行实名制，抓好电信诈骗和网络违法犯罪专项治理，大力开展“雷霆行动”等专项整治，依法严厉打击各类违法犯罪行为，提高突发事件应急处置能力，确保城乡安全、人民安宁、社会安定。

完善治理机制。着力推进完美社区软件建设，全面完善“3+N”治理模式和网格化治理机制，不断提升社区自治、服务能力。鼓励引导社工、志愿者和社会团体发挥作用，增强社会治理合力。推行农村社会治理“3+X”模式，完成村居两委换届选举。落实社会治安综合治理领导责任制，完善立体化社会治安防控体系，打造社会治理信息化、网格化、法治化常德模式。

维护社会稳定。有效推动社区矫正、法律援助、司法鉴定等工作，构建以人民调解为基础的“大调解”体系，积极化解影响社会稳定的突出矛盾，防止经济民生领域风险演变为社会风险。加强和改进信访工作，大力推进阳光信访、法治信访、责任信访，抓好矛盾纠纷源头治理，深入推进领导下访包案和部门联合接访，完善矛盾纠纷多元化解机制，规范和整治信访秩序，使矛盾隐患化解在初始和基层。

四、切实加强政府自身建设

打铁还需自身硬。全面从严治党要求我们必须全面从严治政，进一步加强政府自身建设。

勇于担当，主动作为。为官避事平生耻，狭路相逢勇者胜。只有不怕困难、不怕矛盾、不怕辛苦，才会有所作为。要想干事、会谋事。始终把发展作为第一要务，把事业作为第一追求，将主要心思和劲头放在一心一意谋发展、聚精会神搞建设上。要勇任事、敢担事。没有做不成的事，只有做不成事的人。保持积极进取的精神状态，不回避矛盾，不推卸责任，敢于在加快发展中担重任，敢于在深化改革中涉险滩，敢于在扶贫攻坚中啃硬骨头。要旗帜鲜明地为敢于担当的干部担当，为敢于负责的干部负责。要勤理事、能成事。凡重大决策一以贯之，坚决落地落细，决不动摇折腾；凡重大项目一抓到底，严格督促督查，决不半途而废；凡重大任务一鼓作气，全程问责问效，决不无果而终。

解放思想，创新作为。解放思想的前提是要有思想，创新作为的基础是要有作为。想做就有方法，不做总有借口。政府工作中的大忌是不动脑筋、不思进取。要坚决破除消极的思想障碍，摒弃小富即安的自满、自命不凡的自负、自我膨胀的浮躁，摒弃“伪现代、假开放、浅作为”，让新鲜的思想像空气和阳光一样涌进来。要坚决破除僵化的思维定势，积极探索新常态下做好经济工作的方法和经验，创新发展路径，完善政策举措，着力在优化结构、增强动力、破解瓶颈、补齐短板上取得突破性进展。要坚决破除陈腐的工作惯性，从墨守成规中走出来，从按部就班中走出来，从司空见惯中走出来，提高深化改革、推动发展、化解矛盾、维护稳定的能力和水平，努力摆脱路径依赖、速度情结和换挡焦虑。

优化服务，高效作为。要简政放权，解决活力不足的问题。进一步精简行政审批，取消一切束缚经济社会发展的行政权力，砍掉各类无谓证明和繁琐手续，放开市场主体手脚，用政府权力的“减法”换取市场活力的“乘法”。要从严整治，解决环境不优的问题。坚决向行政执法中的乱检查、乱收费、乱罚款亮剑，坚决向工程建设中的强行阻工、强揽工程、强买强卖亮剑，坚决向办事服务中的索拿卡要、设租寻租、不作为缓作为乱作为亮剑，坚决向公共资源交易和政府性投资项目招投标中的暗箱操作、串标围标、“猫鼠同眠”亮剑，让破坏经济发展环境的人和事在常德无藏身之处、无立锥之地。要创新平台，解决办事不快的问题。整合提升各类服务平台，巩固深化“两集中三到位”和并联审批等制度，推行“互联网+政务服务”，打破部门“信息孤岛”，变“群众奔波”为“信息跑腿”，变群众来回跑为部门协同办，最大限度方便群众、方便企业、方便基层。要完善机制，解决“为官不为”的问题。建立健全推进落实的责任机制、奖勤罚懒的激励机制和常态化的督查问责机制，坚决整肃庸政、懒政、怠政行为，决不允许占着位子不想事、拿着薪水不干事、带着笑脸不办事。

接受监督，依法作为。政府工作的要义在于尊重宪法、尊重法律、尊重人民。要自觉依法履职。带头尊法学法守法用法，严格依照法定权限和程序行使职权、履行职责，切实做到依法治理经济、依法处理问题，全面提高政府工作人员法治意识和依法行政能力。要自觉严以用权。认真执行市委的各项决策部署，严格依法科学民主决策，严格实施“三清单一目录”，严格规范公正文明执法，把权力关进制度“笼子”。要自觉接受监督。主动接受市人大及其常委会的法律监督和工作监督，执行市人大及其常委会的决议决定，认真研究处理各项审议意见，落实好规范性文件备案审查和“三统一”制度，支持和保障市政协履行政治协商、民主监督、参政议政职能，认真办理人大代表建议和政协提案，完善社情民意办理反馈机制，虚心听取和采纳各民主党派、工商联、无党派人士的意见建议，切实推动各类实际问题的解决。

坚守底线，廉洁作为。从政人员一定要有为有不为、知足知不足。贪婪是最真实的贫穷，满足是最实在的财富。要严格廉政建设责任。认真履行“一岗双责”，强化“两个责任”，始终坚持“两手抓”、两手都要硬。要守住艰苦奋斗初心。坚持厉行节约、反对浪费，进一步降低行政成本，确保“三公”经费只减不增。要扎紧廉洁从政篱笆。把纪律和规矩挺在前面，持之以恒推进正风肃纪，坚持长管长严不放松，坚决防止“四风”问题回潮反弹。完善防腐治腐的制度体系，加强对重点领域、重大项目、关键环节、重要岗位权力运行的监督制约，强化行政监察和审计监督，抓好巡视巡察和审计问题的整改，深入推进政务公开，建设统一的公共资源交易平台，不断铲除滋生腐败的土壤。要高悬反腐倡廉利剑。坚定不移惩治各类腐败行为，坚决整治“雁过拔毛”式腐败等侵害群众利益的不正之风，树立风清气正、勤政廉政的良好形象。

各位代表！

实干托起梦想，奋斗铸就辉煌。让我们更加紧密地团结在以习近平同志为核心的党中央周围，在市委的坚强领导下，不忘初心，继续前进，为开创新常德新创业的崭新局面，为实现建设现代化区域中心城市的宏伟目标，为夺取全面建成小康社会的伟大胜利而努力奋斗！
